# A novel method of using transillumination, conjunctival markings and Pascal solid state laser to treat Uveitis-Glaucoma-Hyphema syndrome

**DOI:** 10.1016/j.ajoc.2022.101296

**Published:** 2022-01-20

**Authors:** Baltej Dhillon, Sarah Madison Duff-Lynes, Charles Richard Blake

**Affiliations:** a University of Central Florida, College of Medicine, USA; b University of Florida Ophthalmology Department, USA

**Keywords:** Uveitis, Glaucoma, Hyphema, Glaucoma Treatment, UGH syndrome, Uveitis-Glaucoma-Hyphema, Iridoplasty

## Abstract

**Purpose:**

To report a case of the resolution of Uveitis-Glaucoma-Hyphema (UGH) Syndrome after a solid state 532 nm frequency-doubled neodymium-doped yttrium aluminum garnet (Nd:YAG) laser iridoplasty guided by temporary conjunctival markings.

**Design:**

Interventional Case Report.

**Methods:**

In a case of UGH Syndrome in a 79 year-old male, the decision was made to perform a Nd:YAG laser iridoplasty guided by temporary conjunctival markings along the transilluminated defects created from the IOL haptic.

**Results:**

UGH syndrome in this patient had resolved, with follow-up to seven months.

**Conclusion:**

In an active UGH Syndrome case, prior to decisions for an intraocular surgery, a laser iridoplasty should be considered as this technique has the potential of resolution of the complications of UGH syndrome.

## 1 Introduction

Uveitis-Glaucoma-Hyphema (UGH) syndrome, a disease caused by mechanical irritation of uveal tissues, may occur after intraocular lens implantation. The repeated chaffing of the tissue, often from an anterior intraocular lens (IOL) or misdirected/misplaced posterior chamber IOL or lens haptic, may lead to the breakdown of the blood-aqueous barrier, the release of cytokines and inflammatory markers into the aqueous, and the dispersion of pigment and red blood cells leading to occlusion of the trabecular meshwork and an increased intraocular pressure. Patients suffering from this syndrome often present with blurry vision, pain, and photophobia weeks to years after cataract surgery. A hyphema or microhyphema visualized on slit lamp examination is usually able to lead to the diagnosis.^1^ Chronic inflammation, cystoid macular edema, iris neovascularization, and recurrent hyphemas can continue to decrease vision. Treatment of this syndrome involves both topical medications and various surgical and minimally invasive approaches.^2^

We report a case of a 79-year-old male with UGH syndrome from a posterior chamber IOL in which a novel treatment technique using a solid state 532 nm frequency-doubled neodymium-doped yttrium aluminum garnet (Nd:YAG) laser iridoplasty, guided by temporary conjunctival markings, resulted in complete resolution at seven months follow-up.

## 2 Case presentation

**Fig. 1:**
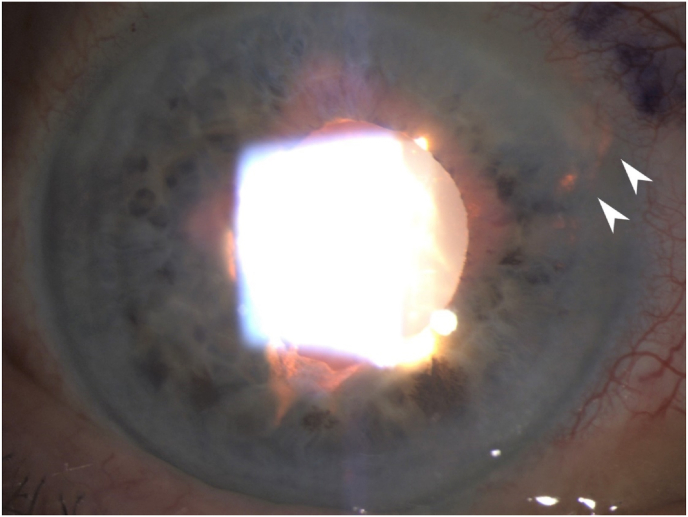
Transillumination defects [White Arrows] where the PCIOL was suspected to be chafing the iris and causing UGH syndrome in this patient.

A 79-year-old man with a history of glaucoma and presumed UGH syndrome in the right eye (OD) was referred to the glaucoma service. He states he was “looking through a film” and had had an episode where his vision worsened significantly two days prior. Best corrected visual acuity (BCVA) was 20/20 OD with a pressure of 18 mmHg OD and 11 mmHg in the left eye (OS). Anterior chamber (AC) exam showed 1–2+ cell, iris sphincter atrophy, and transillumination defects in the superonasal quadrant of the right eye (Fig. 1). No such findings were present in the left eye. Further examination of the posterior chamber intraocular lens showed the haptic to be in the sulcus and rubbing against the iris in the superonasal quadrant. No bleeding was observed from the pupillary margin. The patient was continued on Latanoprost nightly, and started on Dorzolamide/Timolol twice a day OD.

Two days later, the patient presented with significant blurry vision and new onset of floaters OD. He denied flashing lights. On exam BCVA was 20/20–2, IOP was 8 OD and 18 OS, and pupils were round and reactive. Slit lamp examination of the AC showed 4+ red blood cells in addition to the transillumination defects seen previously. Dilated fundus exam OD showed a mildly cupped optic nerve head (0.3h/0.5v), a normal macula, and a flat and attached retina to the periphery 360°. With a new microhyphema in the setting of UGH syndrome, the patient's glaucoma medications were continued and cycloplegic and topical steroid therapy started. Removal of the IOL was unappealing due to remaining good visual acuity and the lengthy time since the previous surgery which would increase the risk of complications. Based on the examination, the IOL haptic was likely irritating the iris superonasally. It was hypothesized that laser application to ablate the bleeding vessels and tighten the iris in that area could thwart further complications from UGH syndrome. Thus, after discussion with the patient, the plan was made to attempt a local laser iridoplasty with the Pascal solid-state Neodymium-doped Yttrium luminum garnet (Nd:YAG).

**Fig. 2:**
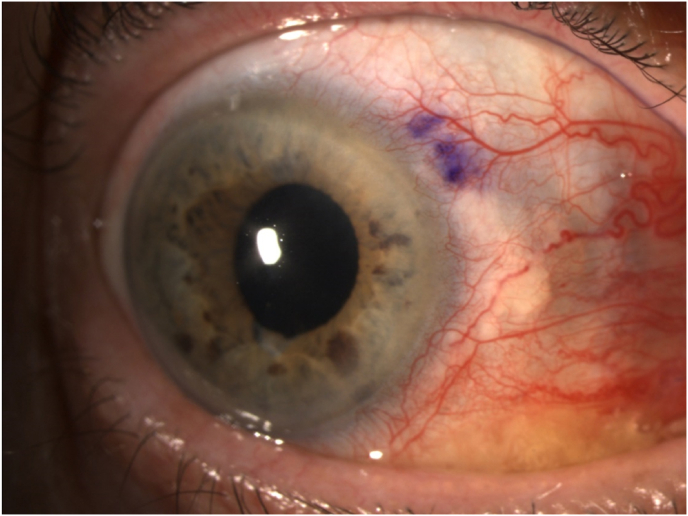
Conjunctival marking near the previously identified transillumination defects made to guide laser iridoplasty for treatment of UGH syndrome.

**Fig. 3:**
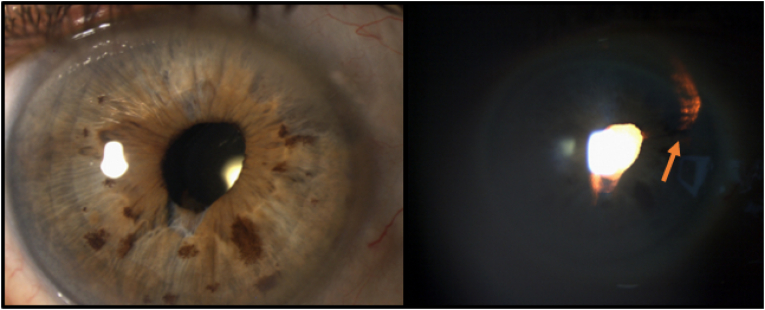
After 7 months, Transillumination defects [Orange Arrow] are visualized at the site of previous laser iridoplasty. (For interpretation of the references to colour in this figure legend, the reader is referred to the Web version of this article.)

The transillumination defects in the iris were identified using a Haag-Streit Slit Lamp model 900 and a surgical marker was used to create a conjunctival marking along the circumference of the affected iris in order to guide the future laser treatment (Fig. 2). The Pascal solid state laser was set to a power of 600 mW and 179 spots of treatment were applied in the affected area, as guided by the markings. The same medical management was continued after the procedure. At follow-up a month later, the hyphema had resolved and the AC was deep and quiet. At that time, all eye medications were stopped except Latanoprost. The patient continued to be followed and at 7 months there had been no further attacks or complications. The appearance of the iris at 7 months is shown in Fig. 3.

## 3 Discussion

Management of UGH syndrome has typically involved topical or systemic medications to control inflammation caused by the release of cytokines due to the mechanical chafing of iris tissue. Although most commonly caused by anterior chamber IOLs,^1^ posterior chamber IOLs, such as in our case, and even glaucoma drainage implants have been indicated in the development of UGH syndrome.^3^ It has previously been suggested that the management of hyphemas in UGH syndrome should simply be activity restriction, head elevation, a cycloplegic drop, and glaucoma medications if necessary.^1^

Further management of UGH involves both invasive and minimally invasive options. IOL explantation is often performed in the setting of significantly reduced visual acuity and increased IOP. Other treatment options include suturing of the IOL to the iris,^4^ suturing the IOL to the sclera, IOL haptic amputation, IOL rotation or manipulation,^5^ placement of a capsular ring to redistribute zonular tension, or serial intracameral anti-vascular endothelial growth factor (anti-VEGF) injections.^6^ While surgical options have the advantage of removing the source of the problem, an initial surgery might not resolve UGH syndrome in as many as 23.4% of cases.^5^ Additionally, there are many potential risks associated with such a surgical procedure, including but not limited to infection, retinal detachment and cystoid macular edema.^1^ Anti-VEGF medications are an appealing treatment alternative as this medication has the ability to manage both the inflammation and abnormal iris vasculature, however, acute and sustained increases in intraocular pressure, possible damage to the corneal endothelium over time, and risk of endophthalmitis complicate this treatment modality.^6^

Use of laser therapy as a treatment modality is not novel. Transscleral cyclophotocoagulation (TS-CPC) has also been reported as one possible management option for UGH.^2^ A report of TS-CPC was able to use ultrasound biomicroscopy (UBM) to show shrinking of the ciliary body after treatment, reducing contact with the IOL.^2^ While TS-CPC has the advantage of simultaneously being able to treat elevated IOP, TS-CPC is not without risks. Endoscopic-guided cyclophotocoagulation has also been performed, but this increases the risk of complications as it is an intraocular procedure.^7^ Walland et al. reported the first instance of UGH syndrome being treated with iridoplasty using an argon laser in a case of UGH secondary to a presumed Polymethyl methacrylate (PMMA) haptic, with resolution at 9 months follow-up.^3^ It was hypothesized that the laser treatment to affected areas allows for a minimally invasive procedure that can ablate bleeding vessels and also reduce contact between the haptic and iris. Additionally, in a case of UGH in the setting of an EX-PRESS shunt, a localized Argon laser iridoplasty shifted the iris away from the shunt tip, resolving the inflammation and hyphema at 1 month follow-up.^8^

A logistical issue with the application of laser is reliably identifying the area of the iris that requires treatment. UBM has been indicated as a possible option to visualize the location of the mechanical irritation.^3^ Conjunctival marking during slit lamp examination in this case was a practical, quick approach to mark the area required for effective treatment. In cases where transillumination defects make it apparent where the source of the mechanical irritation and bleeding is located, marking the conjunctiva at the affected iris is a viable option to guide laser spot placement. Marking the area prior to treatment is important, as the transilluminated area does not show while performing the laser. This is a novel report of using guiding temporary conjunctival markings to complete Nd:YAG laser iridoplasty in UGH syndrome.

Although an interesting method of guiding laser therapy, this type of conjunctival marking is not without its limitations. It requires signs of iris damage on slit lamp exam, such as transillumination defects, to properly identify the site of uveal mechanical irritation. However, if no external defects are seen, UBM may be an alternative to identifying where the mechanical damage is occurring. This could then be followed by a conjunctival marking near where the probe identified the source of the disease.

This technique for treating hyphemas in UGH syndrome is safe and time efficient, and has the ability to prevent the need for a surgical operation. In patients that have likely already had a complicated intraocular surgery previously, this technique is a valuable skill to be considered prior to scheduling a patient for a repeat operation and in carefully selected patients may be able to completely resolve UGH syndrome.

## Note

Grant given to University of Florida from Research to Prevent Blindness.
